# Identification of key genes in atrial fibrillation using bioinformatics analysis

**DOI:** 10.1186/s12872-020-01653-4

**Published:** 2020-08-10

**Authors:** Yueheng Liu, Rui Tang, Ye Zhao, Xuan Jiang, Yuchao Wang, Tianxiang Gu

**Affiliations:** Department of Cardiac Surgery, First Affiliated Hospital, China Medical University, No.155, Nanjing Street, Heping District, Shenyang, 110001 Liaoning China

**Keywords:** Atrial fibrillation, Microarray, Bioinformatics, Pathophysiology

## Abstract

**Background:**

Atrial fibrillation (AF) is one of the most common arrhythmia, which brings huge burden to the individual and the society. However, the mechanism of AF is not clear. This paper aims at screening the key differentially expressed genes (DEGs) of atrial fibrillation and to construct enrichment analysis and protein-protein interaction (PPI) network analysis for these DEGs.

**Methods:**

The datasets were collected from the Gene Expression Omnibus database to extract data of left atrial appendage (LAA) RNA of patients with or without AF in GSE79768, GSE31821, GSE115574, GSE14975 and GSE41177. Batch normalization, screening of the differential genes and gene ontology analysis were finished by R software. Reactome analysis was used for pathway analysis. STRING platform was utilized for PPI network analysis. At last, we performed reverse transcription-quantitative polymerase chain reaction (RT-qPCR) to validate the expression of key genes in 20 sinus rhythm (SR) LAA tissues and 20 AF LAA tissues.

**Results:**

A total of 106 DEGs were screened in the merged dataset. Among these DEGs, 74 genes were up-regulated and 32 genes down-regulated. DEGs were mostly enriched in extracellular matrix organization, protein activation cascade and extracellular structure organization. In PPI network, we identified SPP1, COL5A1 and VCAN as key genes which were associated with extracellular matrix. RT-qPCR showed the same expression trend of the three key genes as in our bioinformatics analysis. The expression levels of SPP1, COL5A1 and VCAN were increased in AF tissues compared to SR tissues (*P* < 0.05).

**Conclusion:**

According to the analyses which were conducted by bioinformatics tools, genes related to extracellular matrix were involved in pathology of AF and may become the possible targets for the diagnosis and treatment of AF.

## Background

Atrial fibrillation (AF) is one of the most common arrhythmia in the world and the incidence of AF rises as the age increases [1–4]. AF could induce palpitation, heart failure and thrombus formation [5, 6] which may impair the quality of life [7] and increase the risk of mortality [8, 9]. Since the mechanism of AF is not clear, it is important to reveal the mechanism of pathogenesis of AF.

The data of gene expression profiles have been increased rapidly in recent years and bioinformatics method, as a powerful tool, has been used to deeply explore the pathophysiological processes. Differentially expressed genes (DEGs) represented the genes whose expression level changed in pathologic condition compared to healthy condition. Alterations of gene expression were involved in the pathogenesis of AF, thus studying the DEGs between AF tissues and sinus rhythm (SR) tissues was necessary. In this study, we used the publicly available gene microarray data to perform de novo analysis and aimed to construct an updated prediction of new genes as biomarkers of AF to supplement currently available data.

## Methods

### Acquisition of data of gene expression profiles

The data of gene expression profiles were acquired by logging in the Gene Expression Omnibus (GEO) database affiliated to National Center for Biotechnology Information (NCBI) (https://www.ncbi.nlm.nih.gov) and editing key word “atrial fibrillation”. Three hundred eight results were shown including 42 series, 3 datasets, 2 platforms and 261 samples with detailed information. Among the 42 series, 29 series belonged to *Homo sapiens*, 9 series belonged to *Mus musculus*, 2 series belonged to *Rattus norvegicus*, 1 series belonged to *Canis lupus familiaris* and 1 series belonged to *Sus scrofa*. We divided the 29 series which belonged to *Homo sapiens* into 2 groups: expression profiling by high throughput sequencing and expression profiling by array and chose the latter group for further research. Microarray data which included RNA content of left atrial appendage (LAA) were selected. According to the criteria mentioned above, five microarray data (GSE79768, GSE31821, GSE115574, GSE14975 and GSE41177) were selected and downloaded. In GSE79768, there were 7 cases of samples from patients with AF and 6 cases of samples from patients without AF. In GSE31821, the number of cases from AF patients and SR patients was 2 and 2, respectively. In GSE115574, the number of patients with AF was 14 and the number of patients without AF was 15. GSE14975 included 5 atrial tissue samples from patients with AF and 5 from patients without AF. Sixteen samples from AF patients and 3 samples from SR patients were included in GSE41177. More detailed information is shown in Table 1.

**Table 1:** Detailed data of GSE79768, GSE31821, GSE115574, GSE14975 and GSE41177

Sequence number of chip	GSE79768	GSE31821	GSE115574	GSE14975	GSE41177
Platform	GPL570	GPL570	GPL570	GPL570	GPL570
Disease	AF	AF	AF	AF	AF
Chip provider	GMRCL, MingChuan Univ	INSERMU1060	Stem Cell Institute, Ankara University	Universitätsmedizin Greifswald	GMRCL, MingChuan Univ
Address	Tau Yau, Taiwan	oullins, France	Ankara, Turkey	Greifswald, Germany	Tau Yau, Taiwan
Research object	Human	Human	Human	Human	Human
Sample type	LAA	LAA	LAA	LAA	LAA
Sample number (used / total)	13/26	4/6	29/59	10/10	19/38
Sample number (AF / non-AF)	7/6	2/2	14/15	5/5	16/3
Time of updating chip	Aug 09 2016	Apr 20 2018	Feb 04 2019	Aug 28 2018	Sep 18 2013

*AF* atrial fibrillation, *LAA* left atrial appendage

### Preprocessing of raw data, screening of differential genes and drawing of volcano plot and heat map

First, GEO Sample (GSM) which stands for LAA was selected. Then the downloaded platform files and series matrix files were transformed by Perl language software 5.28.1. The probe ID in the matrix files was converted into gene symbol by Perl language and saved as a TXT file. After that, five datasets were merged by Perl language and batch normalization was conducted using the “sva” package of the R software. We deleted duplicated genes and values lacking specific gene symbols. DEGs analysis was conducted using the “limma” package of R software. DEGs were selected by using cut-off values of *P* < 0.05 and |log_2_FC| > 0.5, where FC = fold change. Finally, volcano plot was drawn according to the data above and the “pheatmap” package of R software was applied to construct heat map.

### DEGs functional enrichment analysis

On the basis of the DEGs from the merged five datasets, gene ontology (GO) enrichment analysis and pathway enrichment analysis were performed using the “clusterProfiler” package of R software and reactome [10, 11] (https://reactome.org/), respectively. GO analysis is a widely used bioinformatics tool to investigate the annotation of genes and proteins. It can be used to integrate annotation data and provide tools access to all the data provided by the project. Reactome is an open-source, open access, manually curated and peer-reviewed pathway database. The goal is to provide intuitive bioinformatics tools for the visualization, interpretation and analysis of pathway knowledge to support basic and clinical research, genome analysis, modeling, systems biology and education. A q value< 0.05 was identified as significant difference in GO analysis; false discovery rate (FDR) < 0.05 was identified as significant difference in pathway analysis.

### Protein-protein interaction network analysis

We applied Search Tool for the Retrieval of Interacting Genes (STRING) [12] 11.0 (https://string-db.org/) to construct a protein-protein interaction (PPI) network based on the DEGs detected above. Interactions of proteins in the STRING database were screened by using an interaction score criterion. PPI pairs with an interaction score > 0.4 were considered significant in this analysis.

### Reverse transcription-quantitative PCR

From 2017.6 to 2018.2, LAA tissues were collected from 20 AF patients who underwent mitral valve repair and maze procedure and 20 SR patients with left atrial thrombus who underwent mitral valve repair, LAA resection and thrombectomy. The study was approved by the Ethics Committee of the First Affiliated Hospital of China Medical University (NO.2017-69-2) and was carried out in accordance with Declaration of Helsinki. Signed informed consents were obtained from all patients prior to tissue collection.

We selected three key genes for validation using reverse transcription-quantitative PCR (RT-qPCR). Total RNA was extracted with TRIzol Reagent (Invitrogen, USA) according to the manufacturer’s instructions. RNA was converted to cDNA using the PrimeScript™ RT reagent Kit (TaKaRa, Japan). RT-qPCR was performed using TB Green™ Premix Ex Taq™ II (TaKaRa, Japan) and the data were analyzed with LightCycler® 480 Instrument (Roche, Switzerland) and the relative changes were calculated using the 2^-△△Ct^ method. SPP1 primers were: forward 5′- CTGTGTTG GTGGAGGATGTCTGC − 3′, and reverse 5′- GTCGGCGTTTGGCTGAGAAGG -3′. COL5A1 primers were: forward 5′- CGTATGATGACCTCACCTATGG − 3′, and reverse 5′- CGTAGTAGTTCTCGTCAAGGTT-3′. VCAN primers were: forward 5′- ACTGAAACTTCCTACGTATGCA − 3′, and reverse 5′- CTCACAAAGTG CACCAACATAA − 3′. β-actin primers were: forward 5′-CCTGGCACCCAGCA CAAT-3′, and reverse 5′- GGGCCGGACTCGTCATAC-3′.

### Statistical analysis

All data analysis was performed with the SPSS system for statistics (IBM SPSS Statistics 22.0). Data were expressed as mean ± standard deviation. The Student’s t-test was used for comparison between two groups. Differences were considered to be significant at a level of *P* < 0.05.

## Results

### Identification of DEGs between AF and SR LAA tissues

**Fig. 1:**
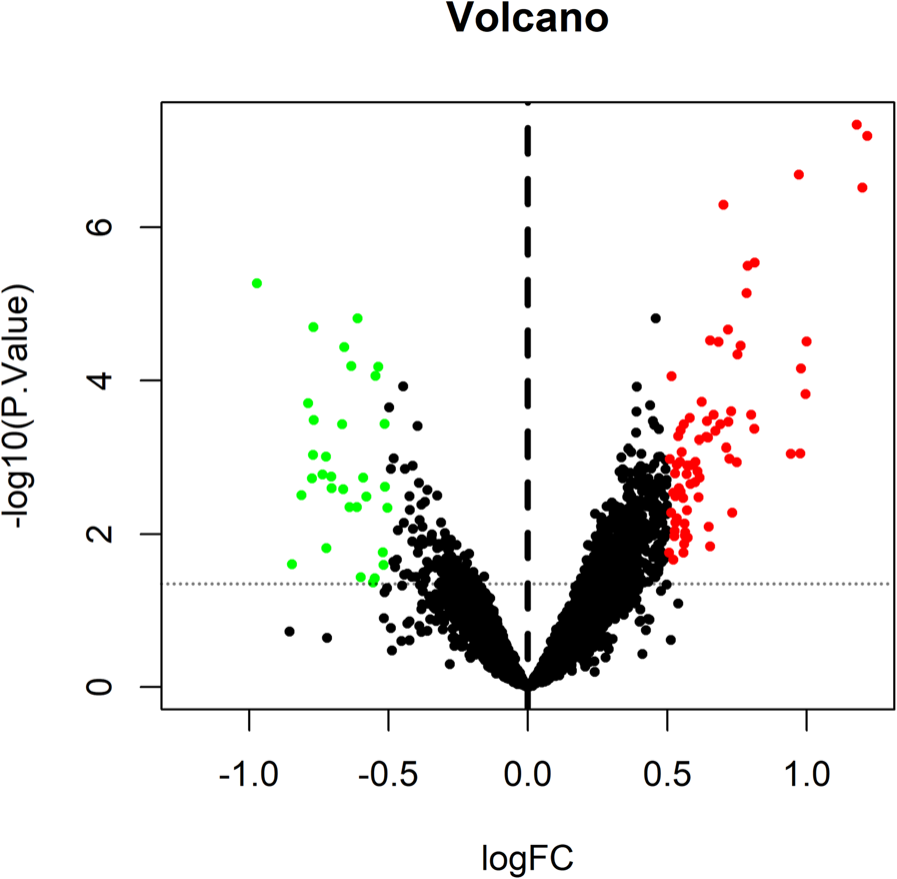
Volcano plot of genes between AF and SR LAA tissues. Genes up-regulated are in red and down-regulated in green. Data points in black mean genes with no significant difference. The differences are set as *P* value< 0.05 and |log_2_FC| > 0.5. The horizontal dotted line indicates *P* = 0.05

**Fig. 2:**
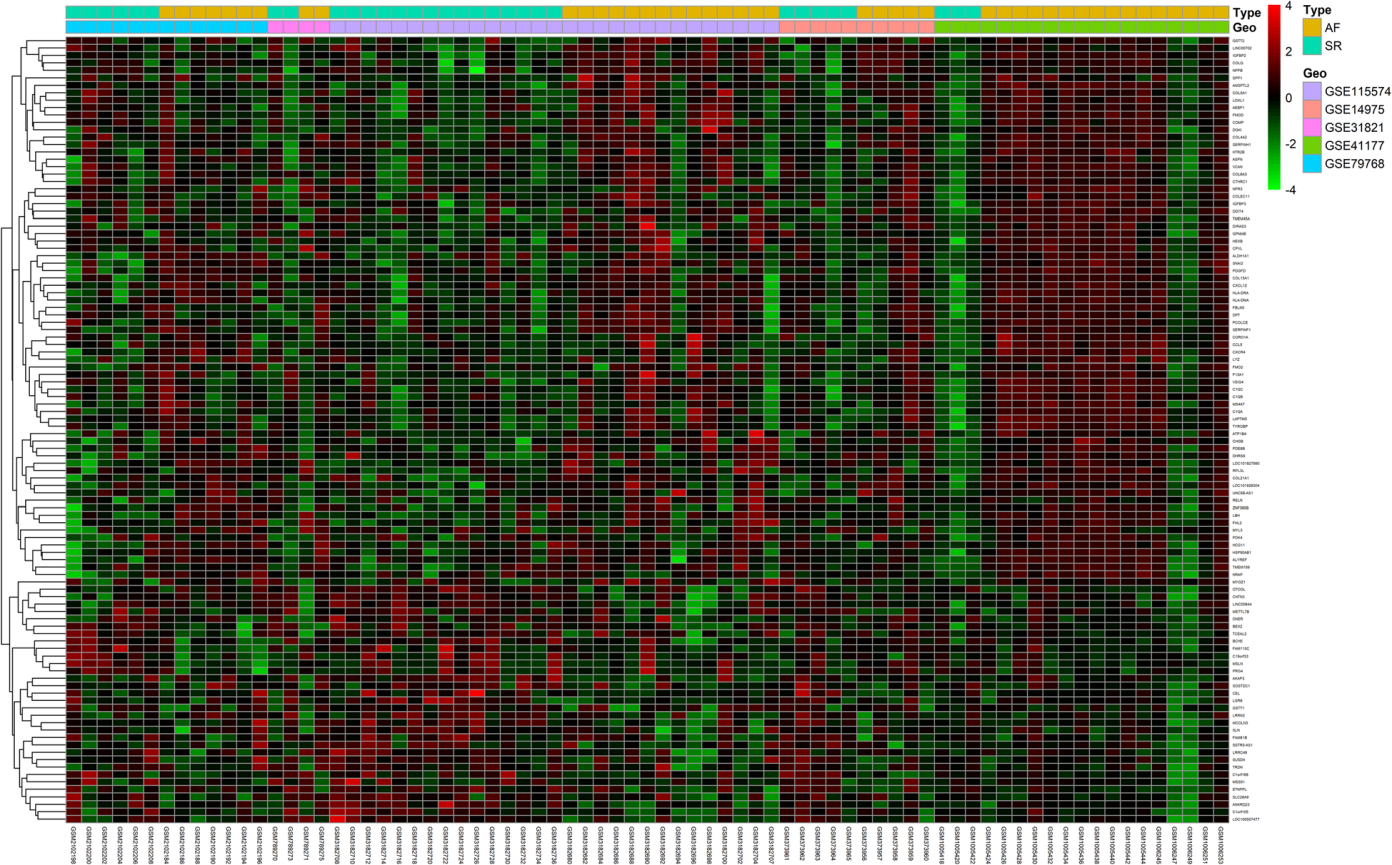
Heat map of the DEGs. Genes up-regulated are in red. Genes down-regulated are in green. Genes expressed at the average level are in black

A total of 106 DEGs were obtained in the merged dataset. Among these DEGs, 74 genes were up-regulated and 32 genes down-regulated. The top 10 DEGs are shown in Table 2. The detailed information about the 106 DEGs is shown in Additional file 1. Volcano plot is shown in Fig. 1 and heat map shown in Fig. 2.

**Table 2:** The top 10 DEGs between AF and SR tissues

Gene	log_2_FC	*P*-value	adj. *P*-value
chromogranin B (CHGB)	1.179275379	4.60E-08	0.000702212
insulin like growth factor binding protein 2 (IGFBP2)	1.217753312	6.49E-08	0.000702212
LBH regulator of WNT signaling pathway (LBH)	0.972408705	2.05E-07	0.001479956
four and a half LIM domains 2 (FHL2)	1.199437818	3.01E-07	0.001628489
angiopoietin like 2 (ANGPTL2)	0.701936948	5.07E-07	0.002196022
snail family transcriptional repressor 2 (SNAI2)	0.813278102	2.88E-06	0.009848093
chromosome 1 open reading frame 105 (C1orf105)	−0.971680865	5.37E-06	0.014526659
collagen like tail subunit of asymmetric acetylcholinesterase (COLQ)	0.784946247	7.24E-06	0.0174026
FYN binding protein 2 (C1orf168)	−0.611589752	1.54E-05	0.030470839
otogelin like (OTOGL)	−0.770019645	2.01E-05	0.035879043

The adjusted *P* value was calculated by false discovery rate (FDR) adjustment

### GO analysis and reactome pathway enrichment

**Fig. 3:**
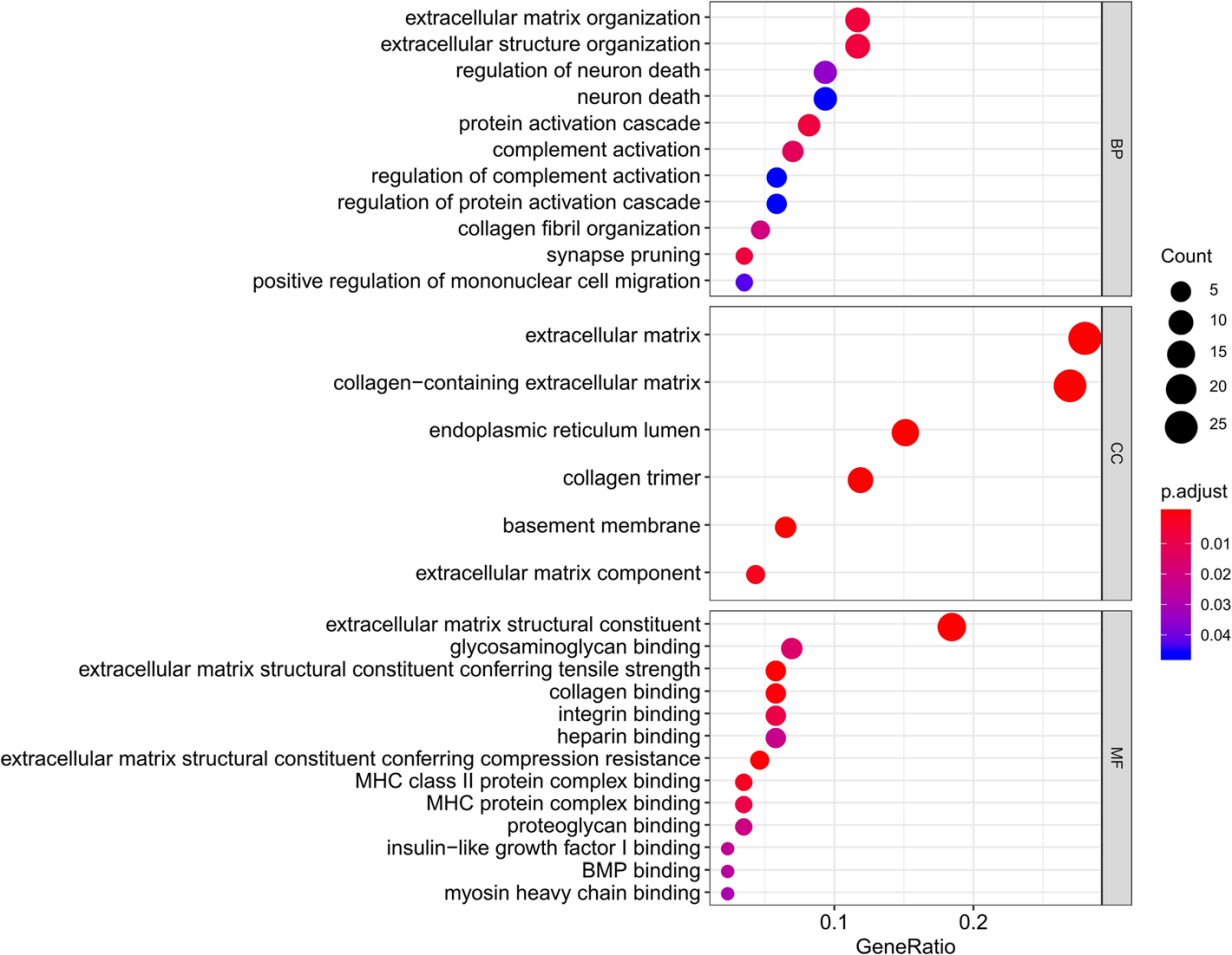
Functional enrichment analysis in GO. The upper part represents biological process; the middle part represents cell composition; the lower part represents molecular function. The words on the left indicates enriched GO, the size of the balls indicates the number of the genes enriched and the color indicates the level of the enrichment

GO analysis of genes includes biological process, cell composition and molecular function. The results are shown in Fig. 3. GO biological process analysis found that DEGs were mainly enriched in extracellular matrix organization, protein activation cascade, synapse pruning, extracellular structure organization, complement activation and collagen fibril organization. In the cell composition part, the DEGs were involved in collagen-containing extracellular matrix, extracellular matrix, collagen trimer, endoplasmic reticulum lumen, basement membrane and extracellular matrix component. In the molecular function section, the genes participated mainly in extracellular matrix structure constituent, extracellular matrix structural constituent conferring tensile strength, extracellular matrix structural constituent conferring compression resistance and collagen binding. The results suggested that DEGs were mostly involved in extracellular matrix structure. We also performed our reactome pathway analysis, which showed DEGs were mainly enriched in Extracellular matrix organization and Collagen formation. All the pathways involved are shown in Table 3.

**Table 3:** Reactome pathway enrichment analysis of DEGs

Reactome pathway	gene number (102)	fold Enrichment	raw *P*-value	FDR
R-HSA-1474244: Extracellular matrix organization	14	9.64	3.45E-10	7.55 E-07
R-HSA-1474290: Collagen formation	8	18.5	2.29E-08	2.51 E-05
R-HSA-1650814: Collagen biosynthesis and modifying enzymes	7	21.51	6.92 E-08	5.05 E-05
R-HSA-3000178: ECM proteoglycans	7	18.96	1.54 E-07	8.44 E-05
R-HSA-2022090: Assembly of collagen fibrils and other multimeric structures	6	20.58	7.91 E-07	3.47 E-04
R-HSA-8948216: Collagen chain trimerization	5	23.39	3.85 E-06	1.41 E-03
R-HSA-186797: Signaling by PDGF	5	18.71	1.06 E-05	3.31 E-03
R-HSA-381426: Regulation of Insulin-like Growth Factor (IGF) transport and uptake by Insulin-like Growth Factor Binding Proteins (IGFBPs)	6	9.96	4.05 E-05	1.11 E-02
R-HSA-216083: Integrin cell surface interactions	5	12.25	7.20 E-05	1.75 E-02
R-HSA-2243919: Crosslinking of collagen fibrils	3	34.31	1.37 E-04	3.00 E-02
R-HSA-8957275: Post-translational protein phosphorylation	5	9.62	2.13 E-04	4.25 E-02

### PPI network integration

**Fig. 4:**
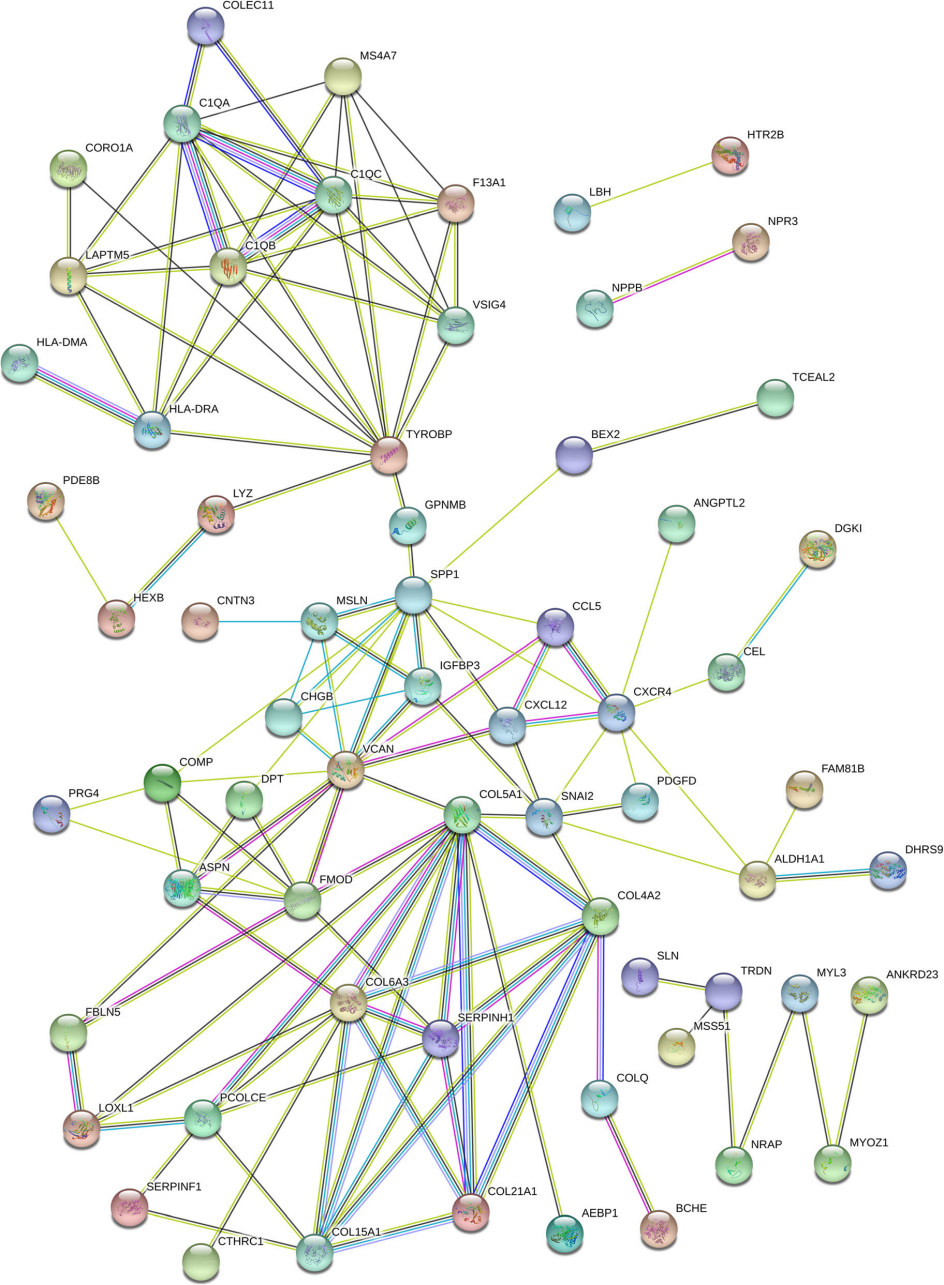
Results of PPI network analysis of DEGs. The balls represent the gene nodes, the connecting lines represent the interactions between genes and results inside the balls represent protein structure. Line colors represent the evidence of PPI

**Fig. 5:**
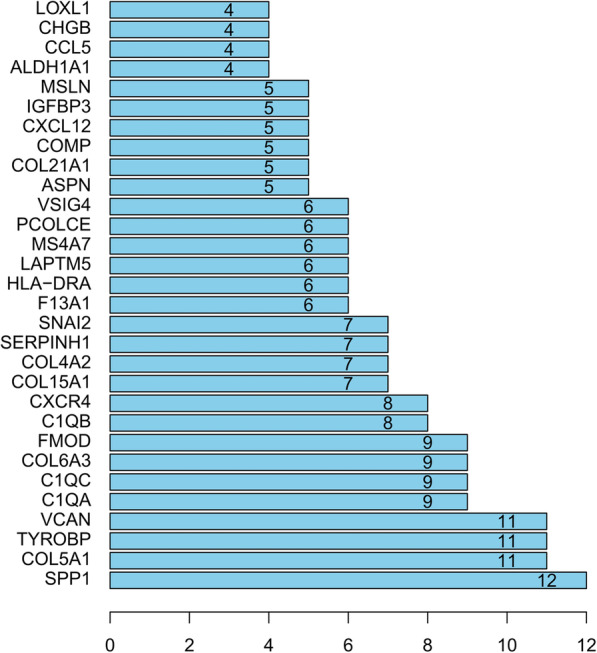
Histograms of core proteins of DEGs. The words on the left indicates gene name and the height of the bar indicates the number of gene connection

We used the STRING database to investigate PPI network, including 74 up-regulated genes and 32 down-regulated genes. Four major proteins were discovered in the PPI network analysis, with secreted phosphoprotein 1(SPP1) connecting 12 nodes, with collagen type V alpha 1 chain (COL5A1) connecting 11 nodes, with versican (VCAN) connecting 11 nodes and TYRO protein tyrosine kinase binding protein (TYROBP) connecting 11 nodes. A PPI network of DEGs and the key proteins were performed as shown in Figs. 4 and 5, respectively. Disconnected nodes in the network were hidden.

### The validation of key genes expression

**Fig. 6:**
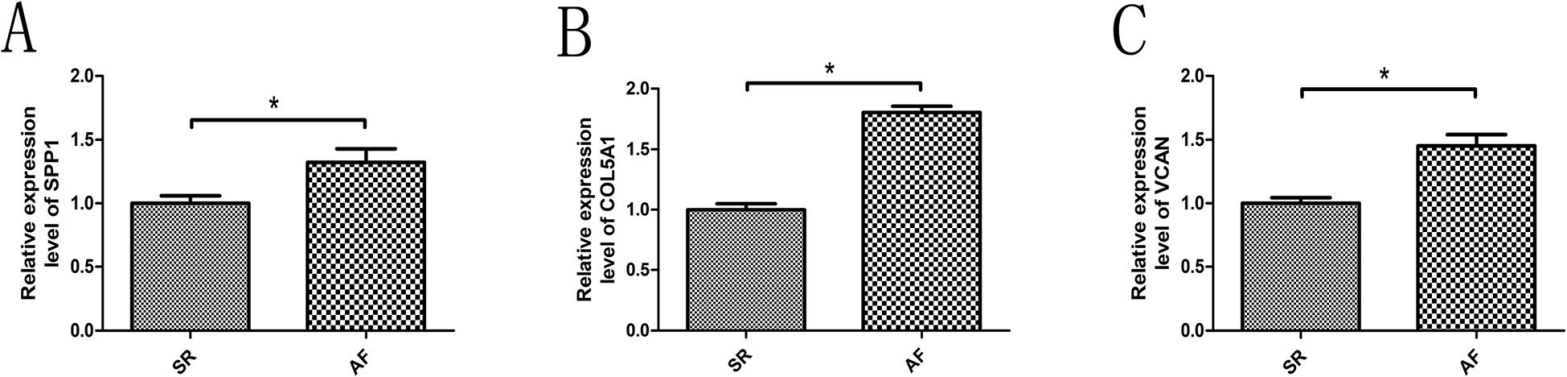
**a** The relative expression level of SPP1 mRNA in SR group and AF group. **b** The relative expression level of COL5A1 mRNA in SR group and AF group. **c** The relative expression level of VCAN mRNA in SR group and AF group. *, *P* < 0.05

We performed RT-qPCR in LAA tissues to further verify the expression of three key genes. As illustrated in Fig. 6, compared with SR group, the transcription levels of SPP1, COL5A1 and VCAN in AF group were significantly increased (*P* < 0.05), which is consistent with our bioinformatics analysis results.

## Discussion

AF is a major medical problem bringing huge burden to the individual and the society [13]. But the treatment of AF is far from satisfactory, so research on the pathogenesis and biomarkers of AF is still necessary and exploring the molecular level dysfunction can provide effective treatment and more diagnostic biomarkers. For example, Wang et al. [14] identified some miRNAs and genes as the target molecules in AF development from two GEO datasets by DEGs screening, construction of regulatory network and GO enrichment analysis. Barth et al. [15] identified key genes in permanent AF from one GEO dataset by integrated method including DEGs screening, hierarchical clustering and GO analysis.

In the present study, we used publicly available microarray data and bioinformatic approaches to predict the potential key genes associated with the pathogenesis of AF. The gene chip data of 5 datasets from GEO were selected and integrated to find a breakthrough point. Using multiple samples and a large amount of microarray data made the experimental results more reliable, providing some valuable references for prevention and treatment of AF. All the samples included were LAA tissue, which avoided the diversity of tissue from different parts. A total of 106 DEGs were obtained. The vast majority of them were up-regulated and the top 10 were CHGB, IGFBP2, LBH, FHL2, ANGPTL2, SNAI2, C1orf105, COLQ, C1orf168, OTOGL.

After that, GO enrichment analysis, pathway analysis and PPI network analysis were applied to analyze the differences between AF and SR patients and investigate the molecular pathogenesis of AF. GO analysis showed that the DEGs mainly participated in extracellular matrix organization, protein activation cascade, extracellular structure organization and collagen fibril organization. These biological processes are important processes involved in the pathophysiological mechanism of AF. Recent research showed that there is a positive correlation between fibrosis and AF, and down-regulation of EZH2 could inhibit Ang-II-induced atrial enlargement and fibrosis and reduce AF vulnerability [16]. Reactome pathway analysis revealed that the DEGs were associated with the extracellular matrix (ECM) organization and collagen formation. More and more studies showed structural remodeling induced by atrial fibrosis and ECM alteration was one of the causes of initiation and perpetuation of AF.

Then, we mapped a PPI network in which the top 3 most influential proteins were SPP1, COL5A1 and VCAN, and they were all enriched in extracellular matrix organization.

SPP1 is involved in the formation and degradation of matrix. Kramerova [17] showed that Spp1 acts upstream of TGFβ to promote fibrosis in x-linked muscular dystrophy (mdx) muscles. Postnatal pharmacological inhibition of Spp1 reduces fibrosis and improves muscle function in mdx mice. Morse [18] demonstrated that in idiopathic pulmonary fibrosis (IPF), macrophages highly expressing SPP1 contribute importantly to lung fibrosis. Ruberti [19] found that SPP1 plasma levels are significantly higher in Primary myelofibrosis compared with essential thrombocytemia and polycytemia vera patients.

COL5A1 is an alpha chain for one of the low abundance fibrillar collagens. Fibrillar collagen molecules are trimers that can be composed of one or more types of alpha chains. Mutations in this gene are associated with Ehlers-Danlos syndrome [20, 21]. Alternative splicing of this gene results in multiple transcript variants. In Sun’s study [22], the collagen-V-knockout stroma demonstrated severe dysfunctional regulation of fibrillogenesis, which indicate central regulatory roles for collagen V in fibril and matrix assembly during tissue development.

VCAN is a member of the aggrecan/versican proteoglycan family. The protein encoded is a large chondroitin sulfate proteoglycan and is a major component of the extracellular matrix. This protein is involved in cell adhesion, proliferation, migration [23] and angiogenesis [24] and plays a central role in tissue morphogenesis and maintenance. Mutations in this gene are the cause of Wagner syndrome [25–27]. Burns [28] found loss of exon 7 in the Vcan gene in mice resulted in ventricular septal defects and altered pulmonary and aortic outflow tracts.

To further validate the expression of the key genes, we tested the LAA tissue by qPCR. We found that the relative expression levels of SPP1, COL5A1 and VCAN showed the same expression trend as in our bioinformatics analysis. There have been some researches on their effects on extracellular matrix. Extracellular matrix alteration, especially fibrosis, is one of the main causes for the development of AF [29]. The DEGs detected in this article were mainly related to the extracellular matrix, which indicated that it is valuable to further study the DEGs and the key genes may become new diagnostic and therapeutic targets for AF.

There are some advantages in our research. First, to the best of our knowledge, our research is the first study to integrate all published microarray datasets related to human AF and LAA mRNA content in the GEO database for bioinformatics analysis. Second, the present study truly reflects the genetic-level changes in the pathophysiological process of AF and can provide possible target molecules for further research. Third, different bioinformatics methods are applied in this research, which can provide more ideas for data analysis. Finally, the chips in this article are published in recent years and all the samples are LAA tissue, which makes the analysis results more accurate and believable. However, the cut-off value we selected in the present study are relatively loose. Further experimental studies are needed to confirm the identified genes and pathways.

## Conclusion

In the merged dataset, we found that SPP1, COL5A1 and VCAN, which are related to extracellular matrix, function together in AF. The present study provided useful information for further exploration of the pathogenesis of AF. Strengthening the research on DEGs of AF will have an important impact on the diagnosis and treatment of the disease.

## Supplementary information

**Additional file 1: Table S1.** The DEGs between AF and SR tissues.

## Data Availability

The datasets generated and/or analyzed during the current study are available in the Gene Expression Omnibus repository and the web links are shown below: https://www.ncbi.nlm.nih.gov/geo/query/acc.cgi?acc=GSE79768 to GSE79768, https://www.ncbi.nlm.nih.gov/geo/query/acc.cgi?acc=GSE31821 to GSE31821, https://www.ncbi.nlm.nih.gov/geo/query/acc.cgi?acc=GSE115574 to GSE115574, https://www.ncbi.nlm.nih.gov/geo/query/acc.cgi?acc=GSE14975 to GSE14975 and https://www.ncbi.nlm.nih.gov/geo/query/acc.cgi?acc=GSE41177 to GSE41177.

## Abbreviations

AF: Atrial fibrillation
DEGs: Differentially expressed genes
LAA: Left atrial appendage
RT-qPCR: Reverse transcription-quantitative polymerase chain reaction
GEO: Gene expression omnibus
NCBI: National Center for biotechnology information
SR: Sinus rhythm
GO: Gene ontology
PPI: Protein-protein interaction
SPP1: Secreted phosphoprotein 1
COL5A1: Collagen type V alpha 1 chain
VCAN: Versican
TYROBP: TYRO protein tyrosine kinase binding protein
ECM: Extracellular matrix
